# Effect of charging conditions on the storage performance of a conformable adsorbed natural gas tank packed with HKUST-1

**DOI:** 10.1371/journal.pone.0336677

**Published:** 2025-12-02

**Authors:** Baifeng Yang, Jiaying Lu, Yixue Sun

**Affiliations:** 1 Institute of transportation and navigation, Quanzhou Normal University, Quanzhou, Fujian, China; 2 Provincial Key Laboratory of Naval Architecture & Ocean Engineering, Institute of Marine Engineering, Jimei University, Xiamen, Fujian, China; Maulana Abul Kalam Azad University of Technology West Bengal, INDIA

## Abstract

Adsorbed natural gas (ANG) technology represents a viable option for energy storage solutions. However, the enhancement of the storage performance is constrained by the thermal effect inherent to conformable ANG storage tanks for small dual-fuel ships. This research had developed a COMSOL numerical model for a 200 L conformable ANG storage tank packed with HKUST-1 to simulate the thermal effects resulting from the charging flow rates and temperature, charging modes. Based on the numerical calculation results, the effects of these charging conditions on the storage performance of the conformable storage tank were analyzed and evaluated. The findings suggested that the heat generated throughout the charging process was a crucial factor affecting both the cumulative storage amount and temperature fluctuations within the ANG system. Furthermore, the study revealed that a combination of low charging temperature and cyclic charging significantly mitigated the adverse effect of thermal effects, thereby improving charging efficiency, and enhancing the overall storage performance of the conformable ANG storage tank.

## 1. Introduction

Natural gas, with its high calorific value, low carbon dioxide emissions and sustainability attributes, is internationally recognized as a clean energy [1]. The utilization of natural gas as a fuel for small dual-fuel ships can reduce carbon emissions, thereby contributing to the mitigation of the greenhouse effect. Natural gas application upon vessels still faces many restrictions [2]. Among these, lack of effective storage technology is one of the crucial technical issues. ANG technology offers the advantage of storing natural gas at low pressures and ambient temperatures [3]. The conformable ANG storage tanks, due to their compact design and ease of use, can be applied as storage tanks for small dual-fuel ships [4]. However, the thermal effect within adsorption system exhibited greater prominence as the charging process progressed, which in turn impedes the improvement of storage performance, and poses challenges for the effective storage of natural gas.

Currently, in order to weaken the thermal effect within ANG adsorbent system, the primary strategies focused on adding heat transfer structures to adsorbent beds and improving the thermal conductivity of the adsorbents. The known heat transfer structures include honeycomb fins [5], 3D printed metal grids [6], and heat transfer fins affixed to the outer wall of storage tanks [7]. Moreover, the structure of conformable ANG storage tanks also served as a measure to enhance heat transfer, facilitating the mitigation of thermal effects in adsorbent beds. As the size of conformable storage tanks increased, the thermal effect of adsorbent beds became evident during the charging process [3]. Nano porous carbon, noted for its high thermal conductivity [8] and MOFs [9,10], characterized by their high specific surface area and pore volume, are regarded as ideal adsorbents for ANG. Adding specific proportions of expanded graphite [5] or graphene oxide [11], etc. into the adsorbents could improve their thermal conductivity. These approaches aimed to improve charging efficiency by facilitating the heat exchange between the interior and exterior of adsorbent beds. Furthermore, the natural gas flow acted as a heat carrier [12], during the cyclic charging, it transferred heat into and out of the adsorbent bed, thereby promoting heat exchange within and around ANG tanks. This mechanism represented another technological approach for managing thermal effects and improving the storage performance. Based on existing literatures [13–15], simulation studies on small-scale ANG storage systems primarily focused on two-dimensional axisymmetric models, which were not applicable to the modeling of a conformable ANG storage tank. Therefore, considering its compact structure, a three-dimensional model needed to be established for numerical analysis.

Given the preceding considerations, it is necessary to construct a three-dimensional model to research the effect of cyclic charging under various conditions on the storage performance of conformable ANG storage tanks. This research focused on a modular, flat-designed ANG storage tank unit. The thermal conductivities of the adsorbent bed in both the longitudinal and transverse directions of its three-dimensional model were calculated. Charging/discharging tests and numerical simulation verification by filling activated carbon SAC-02 (with a BET specific surface area 1550 m^2^·g^-1^) in a vessel with a capacity of approximately 1 L were conducted. Considering the challenges associated with performing tests directly on a 200 L ANG tank in practical applications, the validated model was scaled up to its actual size. Subsequently, HKUST-1 known for its excellent methane adsorption performance, was selected as the adsorbent [6]. A quantitative analysis and evaluation were performed regarding the effects of cyclic charging under different conditions on the storage performance of the conformable tank packed with HKUST-1, focusing on main aspects such as cumulative charging amount, charging duration and efficiency.

## 2. Methodology

### 2.1. Experimental

**Fig 1:**
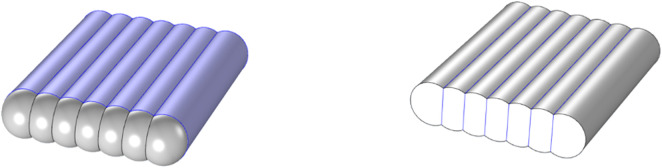
Schematic drawing of a conformable ANG storage system.

**Fig 2:**
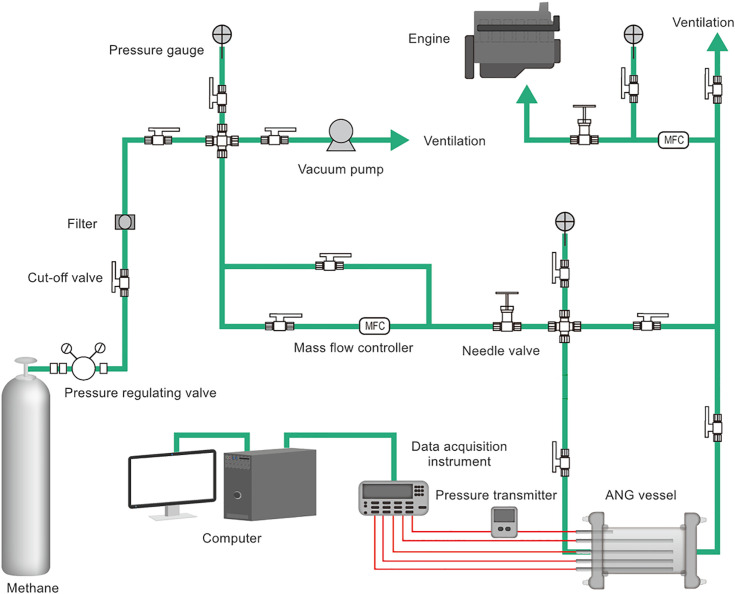
System diagram of the test rig.

**Fig 3:**
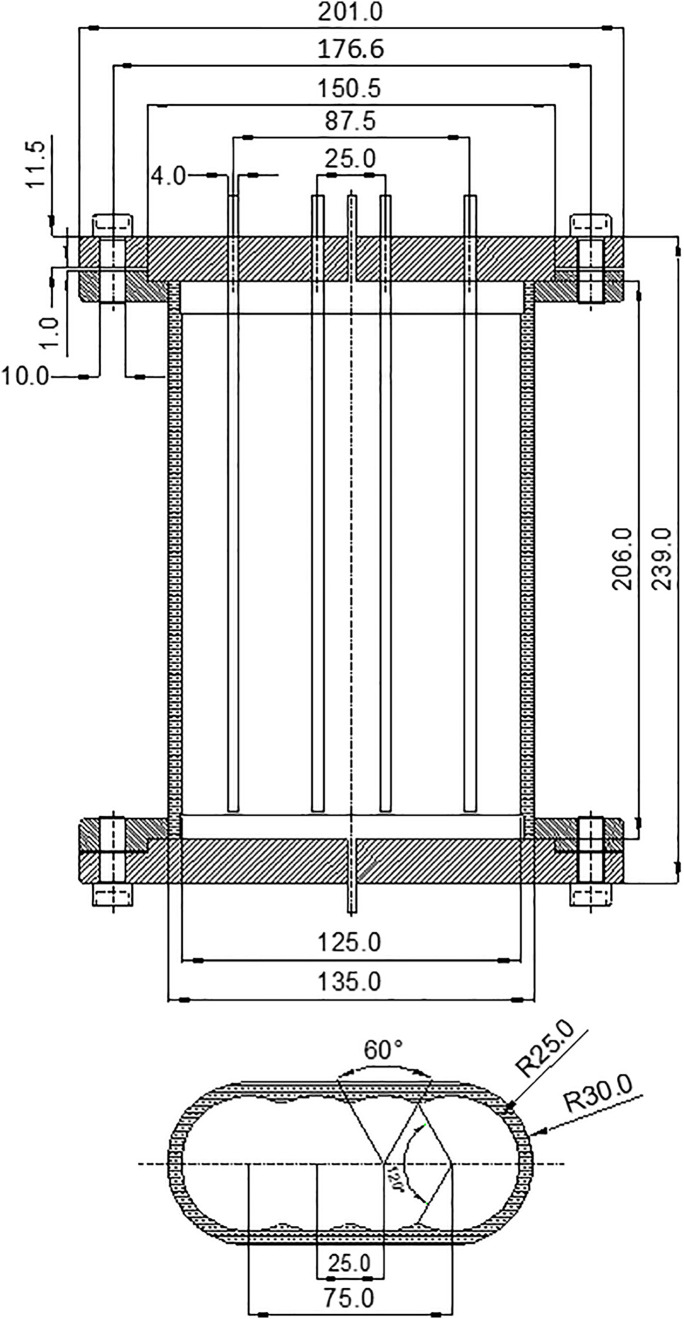
Technical drawing of the storage unit.

The conformable ANG storage tank enhanced the utilization of external space, as illustrated in Fig 1. The test rig for a conformable ANG vessel was presented in Fig 2. In order to provide fuel for about 10 minutes, the vessel with a net capacity of approximately 1L was designed, which constituted a unit of the conformable ANG storage system. Additionally, four T-shaped thermocouples were arranged within the storage vessel to monitor temperature fluctuations at the upper, middle and lower sections, as depicted in Fig 3. Methane, the adsorbate in this study, was a flammable and explosive gas. Prior to conducting the tests, it was necessary to perform leak detection. Nitrogen with a purity of 99.99% was introduced into the system until the pressure reached 3.5 MPa, ensuring that the pressure change within the system was less than 0.2% after 24 h. The coconut shell activated carbon SAC-02 used in this study was produced by Ning de Xin Sen Company in Fujian. After being dried at 150°C for 24 h in a vacuum drying oven, the activated carbon was placed into the ANG vessel. Stainless steel strainers were installed at both ends of the vessel to prevent powder from being expelled. Finally, a pump with a vacuum level of 0.054 Pa was employed to evacuate the system for 12 h, simultaneously removing the impurity gases within the system. Detailed procedures for the tests could be referred to the literature [16].

### 2.2. Numerical simulations

The numerical simulation of the charging process was conducted using COMSOL software. The model equations were discretized utilizing the control volume integral method. The adsorption equilibrium for methane upon the adsorbent could be formulated as


$$\frac{\partial (\varepsilon_{b}\rho_{g})}{\partial t}+\nabla ⬝\left(\rho_{g}\vec{v}\right)=S_{m}.$$ (1)

$$S_{m}=-M_{CH4}\rho_{p}(1-\varepsilon_{b})\frac{\partial n_{abs}}{\partial t}.$$ (2)

The effects of both the viscous resistance and inertial resistance of the methane could be expressed as follows [17]


$$\vec{v}=-\frac{2\nabla P}{\alpha_{E}+\sqrt{\alpha_{E}^{2}+4\beta_{E}\rho_{g}\left\|\nabla P\right\|}},$$ (3)

$$\alpha_{E}=150(1-\varepsilon_{b}{)}^{2}\mu /\varepsilon_{b}^{3}d_{p}^{2},$$ (4)

$$\beta_{E}=1.75(1-\varepsilon_{b})/\varepsilon_{b}^{3}d_{p}.$$ (5)

The energy conservation equation throughout the adsorption and desorption of methane on porous adsorbents could be formulated as


$$(\rho_{g}C_{p}{)}_{eff}\frac{\partial T}{\partial t}+\rho_{g}C_{p}\vec{V}\nabla T-\nabla (k_{eff}\nabla T)=Q+\Phi .$$ (6)

The effective heat capacity of the transient term was expressed as follows


$$(\rho_{g}C_{p}{)}_{eff}=\varepsilon_{b}\rho_{g}C_{pg}+\rho_{b}n_{abs}M_{CH4}C_{pg}+\rho_{b}C_{ps}.$$ (7)

During the adsorption process, assuming that no heat was generated in the adsorbent system, i.e., an isothermal charging process, whose charging efficiency η was 1. The charging efficiency served as a measure of the thermal effect strength of the adsorbent system and η was defined as follows


η=ms/msmd\nulldelimiterspacemd. (8)

The adsorbent bed utilized both longitudinal and transverse thermal conductivities by [18]


$$\lambda_{z}=k_{s}+k_{g}\frac{Pe_{0}}{\text{2}},$$ (9)

$$\lambda_{r}=k_{s}+k_{g}\frac{Pe_{0}}{\text{8}}f\;(R_{i}-r),$$ (10)

$$f(R_{i}-r)=\left\{ \begin{array}{c} \;\;\;{\left(\frac{R_{i}-r}{K_{2}d_{p}}\right)}^{2}for\;0\leq R_{i}-r\leq K_{2}d_{p}\\ 1\;\;\;\;for\;K_{2}d_{p}<R_{i}-r\leq R_{i}, \end{array}\right.$$ (11)

K2=0.44+4e−(Pe0/Pe050\nulldelimiterspace50), (12)

$$Pe_{0}=\vec{v}d_{p}\rho_{g}c_{pg}/k_{g}.$$ (13)

The heat generated throughout the charging process consisted of adsorption heat and compression heat, that’s


$$Q=\rho_{b}\frac{\partial n_{a}}{\partial t}\Delta H-\frac{\text{1}}{\rho_{b}}{\left[\frac{\partial p}{\partial T}\right]}_{p}\cdot T((1-\varepsilon_{b})\frac{\partial p}{\partial t}+\vec{v}\cdot \nabla p).$$ (14)

The modified D-A equation was utilized to compute the excess adsorption amount, that’s [19]


$$n_{exc}=n_{a}-\rho_{g}v_{a}=n_{\max}\exp\left[-{\left[\frac{RT}{\alpha +\beta T}\ln\frac{p_{0}}{p}\right]}^{m}\right]-\rho_{g}v_{a}.$$ (15)

ΔH=αln(nmax/nmaxna\nulldelimiterspacena).m (16)

The mass transfer resistance was ‌characterized by a linear driving force equation, that’s [20]


$$\frac{dn_{a}}{dt}=k(n^{*}-n_{a}).$$ (17)

### 2.3. Model grids division and initial conditions

**Fig 4:**
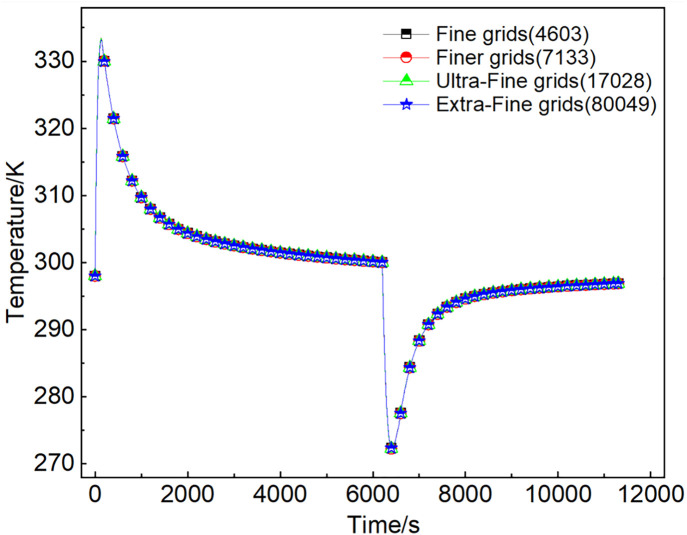
Grids independence verification.

Considering the symmetry of the vessel, a quarter model was established. The COMSOL was utilized to automatically divide the grids. The results of the grid independence study were presented in Fig 4. Four progressively refined mesh divisions were evaluated, revealing that the computed mean temperature curves of the adsorbent bed were essentially coincident. Consequently, in order to reduce computation time, the fine mesh with a grid number of 4603 was selected.

The simulations were conducted based on the following assumptions:(1) The porosity of adsorbent particles was uniform; (2) The gas phase, adsorbent, and adsorption phase were in thermal equilibrium; (3) Methane gas exhibited laminar flow behavior at the storage vessel’s inlet and outlet.

**Fig 5:**
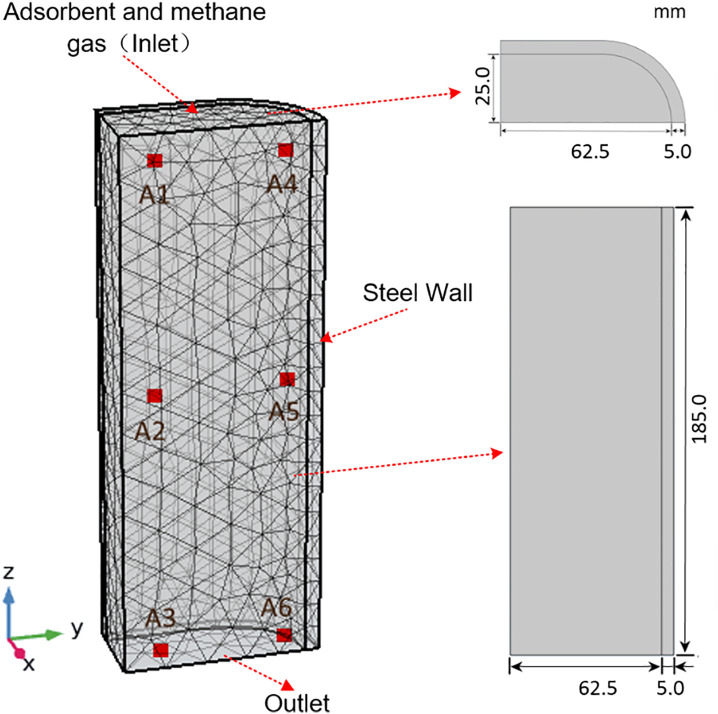
Geometric dimensions and location of monitoring points of the model.

Six points (A1-A6) were set to monitor temperature fluctuations, which were A1 (0, −0.025, 0.17), A2 (0, −0.025, 0.0925), A3 (0, −0.025, 0.003), A4 (0, 0.00625, 0.17), A5 (0, 0.00625, 0.0925) and A6 (0, 0.00625, 0.003), as illustrated in Fig 5. The cyclic charging process was simulated with the following setup. The bottom of the vessel was designated as the outlet, as depicted in Fig 5. Once the pressure within the adsorbent system attained the ‌preset value‌, ventilation commenced at the outlet. At this moment, the adsorbent system maintained the preset pressure, and the charging process was still ongoing. The simulation process was set with a step size of 1 s and a relative tolerance of 1e-4. The initial pressure within the model was 0 Pa, and both the initial and ambient temperature were all 298 K. The density of methane was calculated as an ideal gas, with a specific heat capacity of 2222 J·kg^-1^·K^-1^, a thermal conductivity of 0.0332 W·m^-1^·K^-1^, and a dynamic viscosity of 1.087e-5 Pa·s. These parameters for the vessel wall were 7840 kg·m^-3^, 502 J·kg^-1^·K^-1^, and 13.78 W·m^-1^·K^-1^ respectively.

## 3. Results and discussion

### 3.1. Verification of the numerical model

Methane charging and discharging verification tests were performed using a vessel containing the activated carbon SAC-02. The physical characteristics and fitting parameters for the D-A equation related to the SAC-02 were presented in Table 1. A typical flow rate corresponding to the actual operating conditions of the ship power plant was adopted for model validation tests. The vessel was packed with about 310 g of SAC-02. The experimental data of the flow rate was recorded using a MFC. The fitting sectioned functions for this charging and discharging mass fluxes were presented in Table 2.

**Table 1:** Physical properties and fitting parameters for the D-A equation of the SAC-02 [21].

Items	SAC-02	Items	SAC-02
Density/kg·m^-3^	690	*n*_*max*_/mol·kg^-1^	19.9493
Specific heat capacity/J·kg^-1^·K^-1^	820	*P*_*0*_/MPa	3022.954
Thermal conductivity/W·m^-1^·K^-1^	0.75	*α*/J·mol^-1^	12472.19
Porosity	0.67	β/J·mol^-1^·K^-1^	11.9263
BET specific surface area/m^2^·g^-1^	1507	*m*	2.4936
Mean pore width/nm	0.77		

**Table 2:** Sectioned functions fitted of the mass fluxes for methane.

time/s	‌Mass fluxes/kg·m^-2^·s^-1^
0-155	0.03247 + 3.38064E-4·t-1.70041E-6·t^2^-8.00005E-9·t^3^-2.61893E-11·t^4^
155-6200	0
6200-6215	5.096572942179644E9-3.2821508848599996E6·t + 792.6291682·t^2^-0.085074417·t^3^ + 3.4241999999999998E-6·t^4^
6215-6564	−5034.856140257771 + 3.076676151721315·t-0.0007051047086614999·t^2^ + 7.182705739999999E-8·t^3^-2.74409E-12·t^4^
6564-11300	0

**Fig 6:**
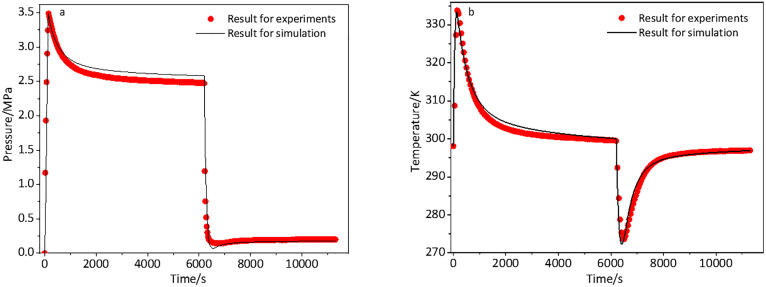
Pressure variations (a) and mean temperature fluctuations (b) of the adsorbent system.

Figs 6a-6b illustrated a comparison of the experimental data with the simulated values regarding pressure variations and mean temperature fluctuations. The simulated values represented the average of results obtained from multiple repetitions of simulations conducted under identical conditions. All mean relative errors remained below 5%, falling within the acceptable limits. This result suggested that the developed numerical model was accurate and appropriate for simulations and predictive researches.

### 3.2. Effect of charging conditions upon the storage performance

The thermal effects of the adsorbent system containing HKUST-1 were evaluated through a numerical model based on a storage tank with a net capacity of about 200 L. Table 3 displayed the physical properties of HKUST-1. The experimental results regarding the excess adsorption of methane on HKUST-1 were analyzed using nonlinear fitting through the D-A equation, and the parameters derived from this equation for HKUST-1 were listed in Table 3.

**Table 3:** Physical properties and fitting parameters for the D-A equation of HKUST-1 [21].

Items	HKUST-1	Items	HKUST-1
Density/kg·m^-3^	587.14	*n*_*max*_/mol·kg^-1^	44.6553
Specific heat capacity/J·kg^-1^·K^-1^	950	*P*_*0*_/MPa	8889.8411
Thermal conductivity/W·m^-1^·K^-1^	0.85	*α*/J·mol^-1^	10492.5
Porosity	0.3	β/J·mol^-1^·K^-1^	23.6792
BET specific surface area/m^2^·g^-1^	1850	*m*	2.7496
Mean pore width/nm	0.7		

#### 3.2.1. Effect of different charging flow rates.

**Fig 7:**
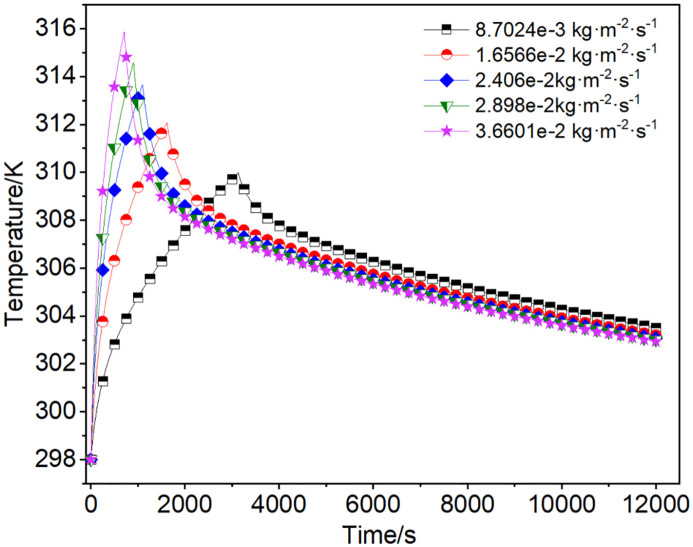
The mean temperature fluctuations within the adsorbent system.

With the rise in charging flow rate, the peak value of the mean temperature rose significantly, and the moment to reach the peak temperature was clearly advanced, so the charging duration to reach the target pressure was shortened in Fig 7. This was mainly due to the charging flow rate increasing, the heat within the adsorbent system couldn’t be transmitted in time, thereby leading to a rapid rise in temperature. From the D-A equation, it could be inferred that the instantaneous adsorption amount would decrease at this time. Therefore, the cumulative charging amount decreased from 1329.6 g to 1259.4 g, reducing around by 5.3%. In contrast, assuming no heat generation during the isothermal charging process, the temperature remained constant, resulting in an isothermal cumulative charging amount of 1468.2 g, which remained unchanged. Since the denominator remained constant while the numerator decreased, the charging efficiency correspondingly declined from 90.6% to 85.8%. Furthermore, under the same target pressure, a higher charging flow rate indicated a shorter charging duration, which decreased by 77.5%, as detailed in Table 4.

**Table 4:** Charging amount and efficiency under different charging mass fluxes.

Mass fluxes/kg·m^-2^·s^-1^	Charging duration/s	Accumulated charging amount/g	Isothermal charging amount/g	Charging efficiency/%
8.7024e-3	3130	1329.6	1468.2	90.6
1.6566e-2	1615	1305.9	1468.2	88.9
2.406e-2	1095	1285.9	1468.2	87.6
2.898e-2	905	1280.1	1468.2	87.2
3.6601e-2	705	1259.4	1468.2	85.8

#### 3.2.2. Effect of different charging temperatures.

**Fig 8:**
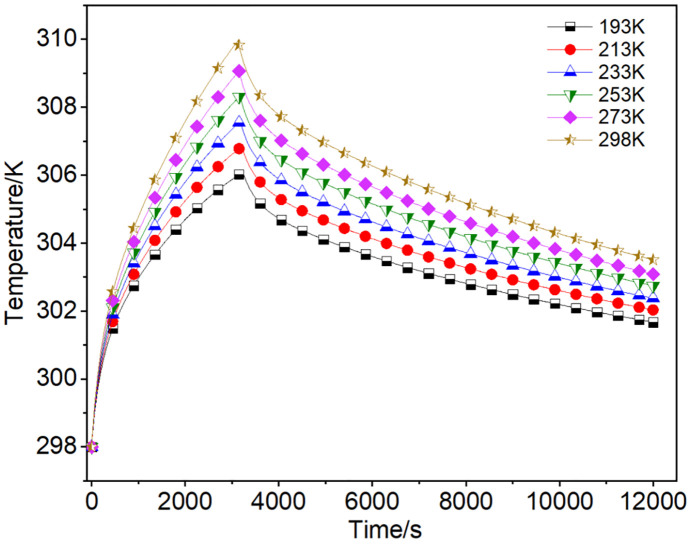
The mean temperature fluctuations in the adsorbent system under various charging temperatures.

Based on the findings from the previous section, it was evident that a mass flux of 8.7024e-3 kg·m^-2^·s^-1^ for charging resulted in both the maximum cumulative charging amount and charging efficiency of the adsorbent bed. Consequently, this section utilized this mass flux for further investigation. When charging at different temperatures, the mean temperature fluctuations exhibited a consistent trend, as shown in Fig 8. The maximal mean temperature within the adsorbent system at a charging temperature of 193 K was about 1.4% lower than that at room temperature, while the cumulative charging amount increased by 3.3% approximately. Due to the same charging flow rate, the time taken for the adsorbent bed to reach the same pressure was essentially consistent across different charging temperatures. A lower charging temperature significantly decreased the mean temperature of the adsorbent bed. According to the D-A equation, this would improve both the accumulated charging amount and isothermal charging amount, while also cause an increase in the temperature difference between the interior and exterior of the adsorbent bed, and lead to a decrease in the charging efficiency. This relationship was illustrated in the data presented in Table 5.

**Table 5:** Charging amount and efficiency at different charging temperatures.

Charging temperature/K	Maximal mean temperature/K	Accumulated charging amount/g	Isothermal charging amount/g	Charging efficiency/%
193	306.1	1374.6	1526.7	90
213	306.9	1365.5	1514.5	90.2
233	307.6	1354.5	1502.1	90.2
253	308.3	1348.0	1491.8	90.4
273	309.1	1339.7	1481.1	90.5
298	310.0	1329.7	1468.2	90.6

#### 3.2.3. Effect of different charging modes.

**Fig 9:**
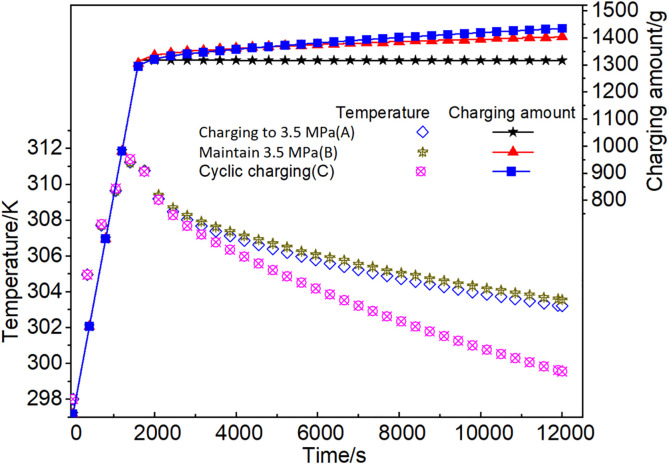
The mean temperature fluctuations and accumulated charging amount of the adsorbent system at different charging modes.

As depicted in Fig 9, after attaining the peak temperature, the mean temperature within the adsorbent system could be decreased rapidly during cyclic charging. After reaching the maximum temperature, the curve exhibited a swift decline to room temperature. During the cyclic charging(C) process, meanwhile after the pressure of the adsorbent bed attaining 3.5 MPa, the cumulative charging amount continued to increase, showing a growth of about 9.0%, which was significantly higher than that of charging mode(A). This was primarily attributable to that the temperature of the adsorbent bed continuously decreased, and methane was constantly adsorbed. Under the charging mode(A), charging ceased once the pressure reaching 3.5 MPa. As a result, the temperature declined faster than other modes, and the cumulative charging amount remained consistent. As cyclic charging(C) at 298 K, the charging efficiency was 96.9%, very close to that of the isothermal charging, the heat of adsorption and compression generated by the adsorbent bed was transferred rapidly by the gas flow, ‌showing that the thermal effects had been weakened to a very low level. In contrast, charging mode (B), characterized by continuous charging without circulation, maintained the pressure at 3.5 MPa, leading to the continuous generation of heat and gradual temperature fluctuations, whose charging efficiency was slightly lower than that of cyclic charging (C), as detailed in Table 6.

**Table 6:** Charging amount and efficiency at different charging modes.

Charging modes	Accumulated Charging amount/g	Isothermal charging amount/g	Charging efficiency/%
Charging to 3.5 MPa (A)	1305.7	1468.2	88.9
Maintain 3.5 MPa (B)	1392.8	1468.2	94.9
Cyclic charging (C)	1423.7	1468.2	96.9

#### 3.2.4. Effect of charging modes combined with different charging flow rates and temperatures.

**Fig 10:**
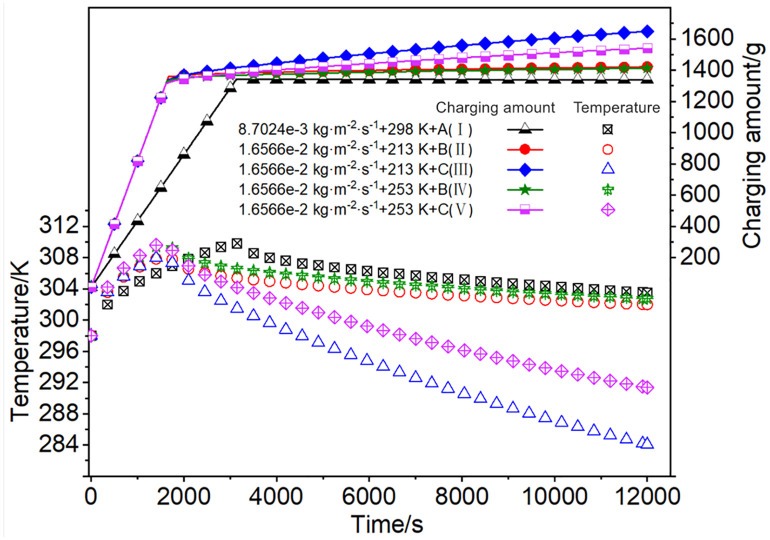
The mean temperature fluctuations and accumulated charging amount within the adsorbent system at charging modes combined with different charging flow rates and temperatures (A-Charging to 3.5 MPa, B-Maintain 3.5 MPa, C- Cyclic charging).

The mean temperature within the adsorption system under the charging condition Ⅲ declined significantly faster than that in other cases, with the corresponding cumulative charging amount reaching the maximum of 1638.3 g, as listed in Table 7. In comparison, when charging under condition Ⅴ, the mean temperature decline was only slower than that under condition Ⅲ. Under condition Ⅰ, the mean temperature was the highest and decreased at the slowest rate. The cumulative charging amount at condition Ⅴ was about 6.6% lower than that at condition Ⅲ. Moreover, the cumulative charging amount at condition Ⅲ was approximately 23.3% higher than that at condition Ⅰ in room temperature, the maximum mean temperature at condition Ⅰ reaching 309.8 K. The fluctuations of the mean temperature and cumulative charging amount at conditions Ⅱ and Ⅳ were relatively close, as shown in Fig 10. The charging flow rate determined the inflection points of each curve in Fig 10. Compared to the charging flow rate, cyclic charging and cryogenic intake were two crucial factors that could significantly reduce the mean temperature, while simultaneously enhancing the cumulative charging amount in the adsorption system, which approaching isothermal charging efficiencies as listed in Table 7.

**Table 7:** Charging amount and efficiency at charging modes combined with different charging flow rates and temperatures.

Charging conditions	Accumulated charging amount/g	Isothermal charging amount/g	Charging efficiency/%
8.7024e-3 kg·m^-2^·s^-1^ + 298K + A (Ⅰ)	1328.7	1468.2	90.5
1.6566e-2 kg·m^-2^·s^-1^ + 213K + B (Ⅱ)	1411.2	1516.8	93.0
1.6566e-2 kg·m^-2^·s^-1^ + 213K + C(Ⅲ)	1638.3	1730.8	94.7
1.6566e-2 kg·m^-2^·s^-1^ + 253K + B(Ⅳ)	1402.9	1496.6	93.7
1.6566e-2 kg·m^-2^·s^-1^ + 253K + C (Ⅴ)	1530.9	1609.6	95.1

**Fig 11:**
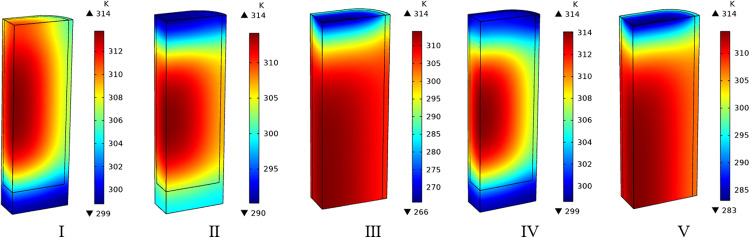
Temperature clouds for adsorbent beds under various charging conditions at the moment charging completed.

Fig 11 demonstrated that at the moment charging completed, these charging conditions had a notable effect on the temperature at the inlet region within the adsorption system, with minimal impact upon that of the center region instead, where temperature was nearly uniform at about 314 K. Over time, compared to other conditions, cyclic charging combined with cryogenic intake could transfer heat and cool the adsorbent bed rapidly, progressively reduce the temperature within the entire adsorption system, achieving the goal of improving charging efficiency.

## 4. Conclusion

COMSOL was utilized to carry out numerical simulations to explore the storage performance of the conformable ANG tank. This research primarily analyzed and discussed the impact of various charging conditions upon the storage performance of the large-scale conformable adsorption system containing HKUST-1. Only methane was considered as the main component of natural gas, whereas in reality, natural gas also contains minor amounts of ethane, propane, butane and so on. The discussion on the impact of low-temperature factors on simulation results in the model was limited. Future researches could focus on the charging characteristics of multi-component natural gas in ANG. The following conclusions had been drawn:

1) Increasing the charging flow rate negatively impacted both the enhancement of cumulative charging amount and efficiency. Specifically, the cumulative charging amount decreased from 1329.6 g to 1259.4 g, representing a reduction of approximately 5.3%. Additionally, the charging efficiency declined from 90.6% to 85.8%.
2) Employing a lower charging temperature could enhance the cumulative charging amount, while resulting in a slight reduction in the charging efficiency. For instance, when using a charging temperature of 193 K compared to the room temperature, the peak of the mean temperature experienced a reduction of about 1.3%, while the cumulative charging amount rose by around 3.4%. Furthermore, as the charging temperature decreased, the charging efficiency of the adsorbent bed exhibited a minor downward trend.
3) Cyclic charging optimized the charging process, making it more close to an isothermal process, significantly mitigating the thermal effect within the adsorbent system, and achieving greater cumulative charging amount and efficiency. Notably, cyclic charging could ‌largely influence the temperature fluctuations in the inlet region of the adsorbent system, while having less effect on that in the central region. The optimal charging conditions were as follows: a flow rate of 1.6566e-2 kg·m^-2^·s^-1^, a temperature of 213 K, and the implementation of cyclic charging, with a maximum cumulative charging amount of 1638.3 g and a charging efficiency of up to 94.7%.

### Nomenclature

**Table:** 

$n_{\max}$	Saturated adsorption amount per unit mass of adsorbent, mol·kg^-1^	Greek α	Enthalpy factor, J·mol^-1^
$n_{exc}$	Excess adsorption amount per unit mass of adsorbent, mol·kg^-1^	β	Entropy factor, J·mol^-1^·K^-1^
$n_{a}$	Absolute adsorption amount per unit mass of adsorbent, mol·kg^-1^	$\rho_{g}$	Density of methane gas, kg·m^-3^
$n^{*}$	Equilibrium adsorption amount, mol·kg^-1^	$\rho_{b}$	Density of the adsorbent bed, kg·m^-3^
$P_{0}$	Pressure of saturated vapor, Pa	v	Specific volume of gas phase, m^3^·mol^-1^
$v_{a}$	Volume of the adsorbed phase, m^3^·kg^-1^	$\varepsilon_{b}$	Adsorbent bed porosity
Q	Heat source term, W·m^-3^	$\rho_{P}$	Density of adsorbent particles, kg·m^-3^
P	Pressure, Pa	$\alpha_{E}$	Coefficient of viscous resistance
T	Temperature, K	$\beta_{E}$	Coefficient of inertial resistance
$M_{CH_{4}}$	Molecular mass of methane, kg·mol^-1^	μ	Dynamic viscosity of methane, Pa·s
ΔH	Isosteric heat of adsorption, J·mol^-1^	$\vec{v}$	Darcy velocity vector, m·s^-1^
$c_{pg}$	Specific heat capacity of methane gas, J·kg^-1^·K^-1^	ω	Acentric factor
$c_{ps}$	Specific heat capacity of adsorbent, J·kg^-1^·K^-1^	Φ	Viscous diffusion term
m	Adsorbent heterogeneity parameter	$\lambda_{Z}$	Longitudinal thermal conductivity of adsorbent bed, W·m^-1^·K^-1^
R	Universal gas constant, J·mol^-1^·K^-1^	$\lambda_{r}$	Transverse thermal conductivity of adsorbent bed, W·m^-1^·K^-1^
$d_{p}$	Mean diameter of adsorbent particle, mm		
k	Mass transfer coefficient		
$m_{s}$	actual cumulative charging amount, kg		
$m_{d}$	charging amount during the isothermal charging process, kg		
$S_{m}$	the mass source term		
$Pe_{0}$	Peclet number		
$k_{s}$	Thermal conductivity of adsorbent, W·m^-1^·K^-1^		
$k_{g}$	Thermal conductivity of methane gas, W·m^-1^·K^-1^		
$R_{i}$	Inner radius of adsorbent system, m		
r	Radial position within the adsorbent system, m		
